# Genitourinary Blastomycosis in a Young Male Patient: A Case Report and Review of Diagnostic Challenges

**DOI:** 10.1155/2023/4713948

**Published:** 2023-12-19

**Authors:** Emily Hillman, Hangcheng Fu, Randa Obid, Uzoma A. Anele

**Affiliations:** ^1^Department of Urology, University of Louisville School of Medicine, Louisville, KY 40217, USA; ^2^Department of Pathology, University of Louisville School of Medicine, Louisville, KY 40217, USA

## Abstract

Disseminated blastomycosis is an endemic fungal infection that rarely manifests with genitourinary involvement. We present a unique case of a 28-year-old professional male gamer with a remote history of hemoptysis and cervical lymphadenopathy who presented with hematospermia, lower urinary tract symptoms (LUTS), and persistent groin abscesses after left orchiectomy at an outside hospital. He underwent drainage of groin abscess and prostate biopsy for an abnormal digital rectal exam which revealed disseminated blastomycosis requiring systemic, long-term antifungal treatment. We have also included a review of literature to note clinical patterns in presentations and highlight the diagnostic challenges that this infection presents.

## 1. Introduction

Blastomycosis is a disease caused by the thermally dimorphic fungus *B. dermatitidis* which is particularly prevalent in the Southwestern United States, Ohio River Valley, and the Great Lakes Region, and thus referred to as an endemic mycosis [1]. Epidemiological information about blastomycosis is derived from investigations of small outbreaks and case series as this disease is rare and is currently reportable in only 6 states and 2 Canadian provinces. As such, the true incidence is unknown and may afflict 1-2 people per 100,000; however, in hyperendemic areas such as the southeastern counties of Wisconsin, U.S., the incidence exceeds over 100 cases per 100,000 people [2]. Exposure to this fungus commonly occurs in proximity to waterways with moist soil and organic debris, and outbreaks have been documented through perturbations of soil by construction and excavation [3]. Aside from environmental risk factors, male sex and outdoor occupation have been noted to be associated with increased susceptibility to blastomycosis [4]. Initial infection commonly occurs through inhalation of conidia into the lungs, where temperature changes induce *B. dermatitidis* to transform into its yeast phase which causes localized pulmonary infection or dissemination of the disease to the skin, bones, genitourinary system, and central nervous system following an incubation period of 4–6 weeks [5].

Blastomycosis infection is highly variable in severity as some patients will experience an asymptomatic subclinical infection or broader symptoms of weight loss, fever, malaise, and fatigue which may create diagnostic challenges [6]. Genitourinary manifestations in systemic infections have been reported to occur in 20–30% of patients with a predisposition for prostatic and epididymal involvement [7]. Patients can present with nonspecific LUTS as well as urinary retention, dysuria, perineal or suprapubic discomfort, hematospermia, hematuria, and induration of the prostate or epididymis [8, 9]. Definitive diagnosis of Blastomyces infection is traditionally performed through culture which may take several weeks or through direct observation of the yeasts from cytologic or tissue sample by histopathology [10]. Treatment of the disease may include surgical exploration, drainage, and systemic antifungal therapy with amphotericin B or itraconazole as first-line antifungal agents [11]. The following case report presents a 28-year-old immunocompetent male patient with disseminated blastomycosis and prostatic involvement which is one of the few case reports in the literature that highlights genitourinary symptomatology of this mycotic infection and provides key insights into the need for high clinical suspicion of this disease.

## 2. Case Report

The patient is a previously healthy 28-year-old male professional gamer living in the Ohio River Valley with a remote history of left epididymoorchitis who initially presented to an outside hospital with hematospermia, worsening left lower abdominal pain, and left orchialgia. Assessment for urinary tract and sexually transmitted infections was negative. Empiric supportive therapies for prostatitis including tamsulosin were ineffective and cystoscopic evaluation was unremarkable. His symptoms progressed for 6 months, culminating in worsened pain and a left scrotal abscess that subsequently ruptured and necessitated an orchiectomy. One month later, he developed pain and swelling on the right side and was readmitted to the outside hospital. CT abdomen/pelvis with contrast suggested prostatitis and the patient was treated with initially oral and then IV antibiotics. Repeat cystoscopy nearly 1 month later was again unremarkable.

**Figure 1:**
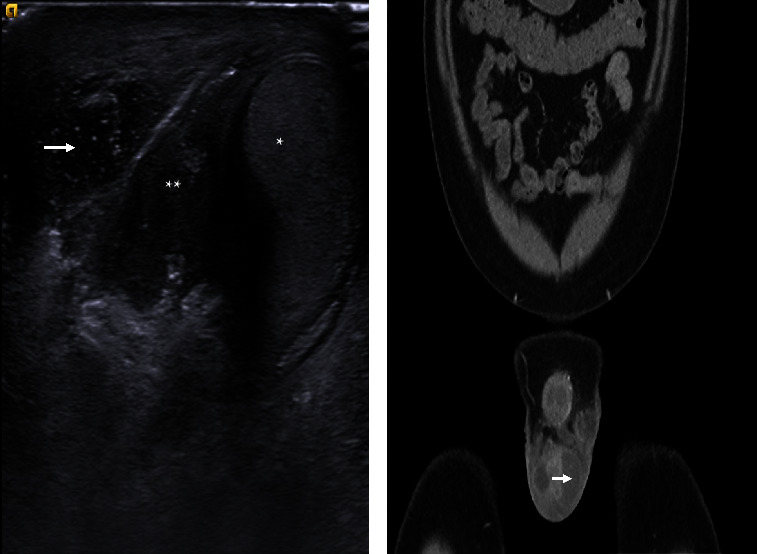
Diagnostic imaging. (a) Scrotal ultrasound depicting hypoechoic 5.5 cm × 3.6 fluid collection (i.e., abscess; arrow) adjacent to epididymis (double star) and testis (star) in the right hemiscrotum. (b) Coronal CT abdomen/pelvis with contrast depicting scrotal abscess formation as suggested by presence of rim-enhancing fluid collection (arrow).

The patient then presented to our institution with symptoms of chronic dysuria, body aches, ostealgia, and 1 week of night sweats. He disclosed that he previously experienced an episode of hemoptysis and left anterior ear lymphadenopathy along with a left forehead wound and skin infection. Physical exam revealed a very firm and enlarged but nontender right testis, and a left hemiscrotum with surgically absent testis and approximately 4 cm raised ballotable area of tenderness along the medial aspect. Additionally, digital rectal exam demonstrated an enlarged approximately 25 g prostate with nodular firmness on the right side. Same-day hospital admission was elected for further investigation and initiation of empiric IV piperacillin/tazobactam. A scrotal ultrasound was performed and showed a 5.5 cm × 3.6 fluid collection in the right hemiscrotum, and a subsequent contrast CT abdomen/pelvis confirmed abscess formation as evidenced by the presence of a rim-enhancing fluid collection (Figure 1). Relevant laboratory workup revealed a PSA of 2.05 ng/mL, WBC of 12.9 × 10^3^/*μ*L, and no growth on urine culture. The following day, he underwent incision and drainage of a right scrotal abscess, excision of a suture granuloma from the left groin, as well as transrectal ultrasound (TRUS) with prostate and seminal vesicle biopsy and tissue cultures. After clinical improvement and recovery from the procedure, he was discharged home on empiric amoxicillin/clavulanic acid.

**Figure 2:**
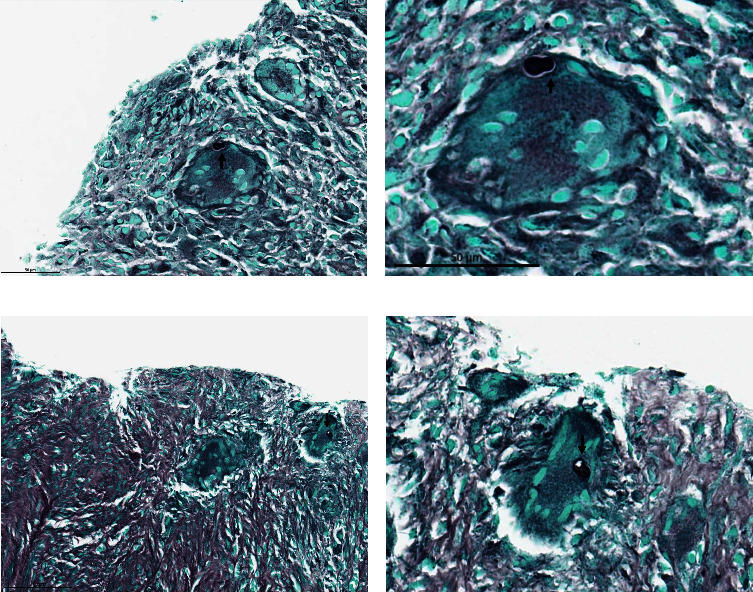
Grocott's methenamine silver stain of prostate and seminal vesicles biopsy samples which demonstrate granulomatous inflammation with necrosis, rare fungal budding yeast forms (arrow). (a) Prostate gland 20x magnification. (b) Prostate gland 40x magnification. (c) Seminal vesicles 20x magnification. (d) Seminal vesicles 60x magnification.

Pathology specimens from scrotal, seminal vesicles, and prostate tissue samples demonstrated granulomatous inflammation with necrosis and rare fungal yeast forms (Figure 2). These findings correlated with intraoperative cultures which were also positive for fungus. Further tissue staining techniques revealed round structures within giant cells which were consistent with *Coccidioides* or *Cryptococcus* species but could not readily differentiate between the two. Additional PCR analysis performed at an external institution detected *Blastomyces dermatitidis* which was also eventually confirmed from the abscess fluid culture. Upon diagnosis of disseminated Blastomycosis, he was referred to an infectious disease specialist for further workup and initiation of long-term itraconazole therapy. At 3 months follow-up, CT of the chest with contrast did not demonstrate any concerning pulmonary disease. However, fungal complement fixation with Blastomyces antibodies by enzyme immunoassay was positive with a titer of 3.3. On the most recent follow-up at 6 months, he reported overall clinical improvement and resolution of dysuria and penoscrotal pain. However, he endorsed obstructive LUTS and anejaculation. Subsequent cystoscopic evaluation demonstrated a prominent verumontanum but otherwise did not reveal any clear signs of obstruction. He was offered a transurethral intervention but preferred observation.

## 3. Discussion

Delays in diagnosis are often experienced as blastomycosis has been named the great mimicker, particularly when urologic manifestations of the disease are pursued through the lens of benign prostatic hyperplasia (BPH), neoplasm, or bacterial infection instead of fungal origin. In one large retrospective study from Mississippi, blastomycosis was suspected at initial patient evaluation in only 18% of 123 patients. Moreover, in nearly half of the patients, the diagnosis was made more than 30 days into their illness [12]. Case reports on blastomycosis in the literature, which are listed in Table 1, illustrate a 2-week to 4-month delay in diagnosis and clinical features similar to those experienced by our patient [9, 13–17]. Our patient exhibited an initial episode of cutaneous lesion, respiratory symptoms, and lymphadenopathy, which progressed to chronic fatigue and bone pain. His genitourinary concerns manifested approximately 3 months later, suggesting the dissemination of blastomycosis. Ultimately, from his initial presentation to an outside hospital, 8 months elapsed before the source of his illness was identified. The extended interval highlights the insidious nature of this mycotic infection as our patient was treated with multiple courses of antibiotics for presumed bacterial prostatitis and scrotal abscesses. Seo et al. reported on a patient with chronic epididymitis and prostatitis whose symptoms of nocturia, hematospermia, and perineal pain persisted over 6 weeks despite repeated antibiotic treatments, and who was diagnosed with blastomycosis infection following prostatic biopsy and culture [9]. Therefore, we propose that fungal infection should be considered, particularly when symptoms are refractory to an antibiotic treatment period of over 4–6 weeks duration.

Investigating patterns of clinical presentation is necessary in decreasing the overall mortality and morbidity of blastomycosis. The mortality rate associated with blastomycosis is reported to be between 4% and 6% and can dramatically increase to 89% with disseminated disease, specifically due to acute respiratory distress syndrome (ARDS) [18, 19]. Bergner et al. reported on a patient with a 2-month history of prostatism who expired 4 days after he presented with acute urinary retention, a distinct change from his previously stable genitourinary complaints which culminated in fatal ARDS [13]. In addition, Gandam Venkata et al. described a patient with a previous history of BPH who also presented with acute urinary retention and expired following hospital admission due to blastomycosis-induced ARDS [14]. A key similarity between the above cases is that the chronic symptoms were followed by a discrete change in clinical status in the form of acute urinary retention or heightened pain. This not only represents a sign of a worsening outcome but may also characterize a pattern of blastomycosis that warrants increased suspicion for clinicians.

Diagnosis of blastomycosis often relies on a clinician's degree of suspicion which can be enhanced by thorough history taking. It is crucial to elucidate a patient's recreational activities such as hunting, fishing, travel to endemic regions, and exposures at home and work especially if there is proximity to construction or decaying wood and water sources [4, 20]. Najdawi et al. reported on a case of genitourinary blastomycosis whose infection was initially treated as bacterial prostatitis, epididymitis, and scrotal abscess for over 4 months until it was elicited that the patient's previous employment 6 months prior was at a construction site in Wisconsin, a U.S. state that is highly endemic for this mycotic infection [21]. Similarly, through a detailed interview with our patient, we learned of his occupation as a professional gamer and his extended time in the basement of his home in the endemic region of the Ohio Valley River, which we believe was the source of his exposure to *B. dermatitidis*. Attics and basements of homes may have contact with decaying organic debris, mechanical soil disruption, and air currents that carry aerosolized conidia, which mimic optimal growing conditions for this fungus [4]. Roy et al. performed an epidemiologic outbreak investigation in Wisconsin, U.S. that occurred in a specific geographic cluster of households and found that there was no common human outdoor or recreational activity associated with the cases [22]. This meant that blastomycosis infection was likely due to the actual residence, a hypothesis that was investigated by Anderson et al. who sampled two of the homes in the previously mentioned study and discovered Blastomyces DNA in air samples. Evidence demonstrated that the basement environment was a suitable growing environment for this fungus and that both the condition of the residence and neighborhood were risk factors in the outbreak [23].

As blastomycosis is a rare disease, it is often not clinically considered which leads to delay in diagnostic culture and treatment. Consequently, patients may suffer unnecessary medical procedures, as did our patient, who underwent an orchiectomy at an outside hospital under the assumption of bacterial infection when his true diagnosis was of a fungal scrotal abscess caused by disseminated blastomycosis. Sloan et al. discussed a case of a 59-year-old male with urinary retention and LUTS who worked as a gardener in Nevada, U.S., and presented with a prostatic abscess. Similar to our patient, he required operative intervention of incision and drainage for adequate source control in addition to antifungal treatment [17]. Extrapulmonary manifestations of blastomycosis may require more than systemic therapy, specifically surgical management, and prompt tissue/fluid culture acquisition is crucial to establish a diagnosis. Despite the prolonged duration of time required for results, a positive culture is an accurate and definitive way to diagnose blastomycosis and should be sought in every case [3]. In addition, diagnosis may be made by direct visualization of the organism in cytologic or tissue samples that exhibit characteristic multinucleate single, broad-based budding yeasts [24]. This has been the most utilized method for rapid diagnosis of blastomycosis [25]. Recently, PCR technology has been developed and is not only accurate with a high specificity and sensitivity but also a time-saving method for blastomycosis detection. The limiting factor with this nucleic acid amplification technology is its lack of commercial availability; however, if institutions can access PCR testing, it is especially useful for the detection of DNA in tissue samples as well as bodily fluids [5, 26, 27].

Although urine antigen tests are available and quick, these have a high cross-reactivity with histoplasmosis, another endemic mycosis, thus incurring a low specificity [28]. Antigen tests are better suited to follow the response to treatments of patients with blastomycosis who are on long-term antifungals as their tests will report decreasing levels of the antigen as the infection is cleared [29]. However, our patient was followed during fungal treatment and postsurgical intervention by immunoassays that targeted *B. dermatitidis* antibodies. This method is similar in efficacy to antigen tracking but is not widely available for clinicians to order [30]. Finally, treatment guidelines emphasize that all patients with blastomycosis should receive antifungal therapy as this disease has risks of recurrence and progression to dissemination. Treatment with itraconazole is the preferred azole for initial therapy of patients with mild to moderate disease and should be conducted for 6–12 months, given that ketoconazole has been demonstrated to be less effective. More severe disease which includes CNS manifestations requires initial treatment with amphotericin B and then itraconazole as step-down therapy [11].

## 4. Limitations

Despite our observations, there are limitations. The generalizability of this case report is limited by its nature as being retrospective and from a single center. In addition, it inherently cannot generate information on the rates, ratios, incidence, or prevalence of this disease process. Therefore, it is only able to provide limited epidemiologic insights to similar Ohio River Valley states due to the lack of required reportability.

## 5. Conclusion

Overall, clues that may aid in the diagnosis of blastomycosis infection include chronic genitourinary complaints with acute changes, prolonged ineffective antibiotic treatment, and a detailed patient interview to determine exposure risk. Due to the reported growth in endemicity and incidence of Blastomycosis, expanded monitoring should be adopted with required disease reporting [31]. Furthermore, this may facilitate an improved epidemiological understanding of this infection and awareness for diagnostic consideration. Moreover, a large multisite study within endemic regions evaluating symptom progression in patients diagnosed with blastomycosis would provide significant insight into the clinical manifestations and patterns of this disease to improve diagnosis and care.

## Figures and Tables

**Table 1:** Review of blastomycosis case reports with genitourinary involvement.

Author/Years	Genitourinary presentation	Time to antifungal initiation	Diagnostic method	Antifungal treatment duration	Outcome
Bergner et al. [13]	LUTS, malaise, chronic weakness, fever	2 months	Postmortem histology, antemortem culture	N/A	Deceased

Gandam Venkata et al. [14]	Acute urinary retention	2 weeks	Culture	N/A	Deceased

Inoshita et al. [15]	Patient 1: Dysuria, acute urinary retention Patient 2: Frequency and burning urination	Patient 1 : 6 weeks Patient 2 : 3 weeks	Microscopic prostatic secretion, histology of prostate	Patient 1 : 3 months IV amphotericin Patient 2: Ketoconazole 1 month	Recovered

Najdawi et al. [21]	Bilateral testicular pain, dysuria, urgency, acute urinary retention	4 months	Culture	Itraconazole 6 months	Recovered

Neal and Nikolai [16]	Urgency, frequency, dysuria, nocturia	6 weeks	Fine needle aspiration	Itraconazole 6 months	Recovered

Sloan et al. [17]	Prostatitis, acute urinary retention, night sweat, fever	>2 weeks	Urine culture, histology of prostate	Itraconazole 1 year	Recovered

Seo et al. [9]	Nocturia, hematospermia, perineal pain	>6 weeks	Staining of histological specimen, culture	Ketoconazole 1 year	Recovered

LUTS = lower urinary tract symptoms.

## Data Availability

No underlying data were collected or produced in this study.
